# Promoting Innovation in State and Territorial Maternal and Child Health Policymaking

**DOI:** 10.1007/s10995-023-03779-1

**Published:** 2023-10-04

**Authors:** Sanaa Akbarali, Ramya Dronamraju, Jessica Simon, Amani Echols, Stacy Collins, Betsy Kaeberle, Atyya Chaudhry

**Affiliations:** 1 Association of State and Territorial Health Officials, 2231 Crystal Drive, Suite 450, Arlington, VA 22202 USA; 2 Association of Maternal and Child Health Programs, 1825 K Street NW, Suite 250, Washington, D.C 20006 USA

**Keywords:** Perinatal Substance Use, Substance Use Disorder, State and Territorial Health Department, Learning Community, Title V MCH

## Abstract

**Introduction:**

The Association of Maternal & Child Health Programs (AMCHP) and the Association of State and Territorial Health Officials (ASTHO) launched the PRISM (Promoting Innovation in State and Territorial MCH Policymaking) Learning Community, funded by the U.S. Department of Health and Human Services (HHS), Health Resources and Services Administration (HRSA), Maternal and Child Health Bureau (MCHB). The goal of PRISM was to build state and territorial health agency program and policy-making capacity to address substance use and mental health in the maternal and child health (MCH) population. Expanding access to care and treatment for perinatal substance use disorders (SUD) emerged as the issue of greatest need for state teams.

**Methods:**

The PRISM Learning Community consisted of three major components: (1) intensive capacity building for cross-agency state teams, which involved action planning, peer-to-peer learning, and technical assistance; (2) programming to inform the MCH field broadly about innovations in perinatal SUD policy and practice; and (3) a program evaluation involving pre-, mid-, and post-assessments and follow-up key informant interviews with state teams. This manuscript is not based on clinical study or patient data, therefore IRB approval was not required.

**Results:**

States reported that their knowledge of perinatal SUDs increased and their cross-agency partnerships were strengthened as a result of their participation in PRISM. States identified four key priorities for their continued work: to improve multisector collaborations, to institute equitable SUD screening practices for pregnant people, to strengthen the perinatal behavioral health workforce, and to enhance Medicaid coverage for perinatal SUD prevention and treatment services. The need to respond to urgent demands of COVID-19 and the stigma associated with perinatal SUDs were the most significant barriers to advancing state action plan goals.

**Discussion:**

Since 2018, the PRISM project has supported nine jurisdictions across two cohorts. Participation in PRISM advanced state policies and programs to improve perinatal SUD care through capacity building, technical assistance, and virtual programming. Findings and lessons learned from PRISM may inform the activities of other states seeking to address perinatal substance use disorders.

## Significance

*What is Already Known About this Subject?* The learning community model is known as an effective method of engagement for adult learning. Learning community models are currently being implemented for a variety of public health issues. Perinatal mental health and SUD care are widely studied from a clinical perspective and have gained national attention as a public health priority.

*What does this Study Contribute to the Existing Body of Knowledge?* This report from the field describes state and territorial programmatic and policy efforts to improve the quality and accessibility of SUD care for perinatal populations through the PRISM Learning Community. Other jurisdictions may find the program and policy options helpful as they advance initiatives in their states to improve maternal and child health.

## Introduction

### Impact of Perinatal Substance use Disorder on Maternal and Child Health Populations

Substance use, particularly the use of opioids, during the perinatal period is an ongoing and urgent public health concern. Women are at increased risk throughout their reproductive years (18 to 44) for developing a SUD; the highest risk occurs between ages 18 to 29 years old (Oh et al., 2017). Mirroring the broader opioid crisis in the United States, the incidence of substance use during pregnancy continues to rise. Data show that maternal-opioid use has increased from 3.5 cases per 1,000 delivery hospitalizations in 2010 to 8.2 cases per 1,000 delivery hospitalizations in 2017 (Hirai et al., 2021). Untreated substance use during the prenatal period is associated with increased risks for obstetric and medical complications, including poor fetal growth, preterm birth, stillbirth, and neonatal abstinence syndrome (NAS) (Haight et al., 2018). For women who are abstinent during pregnancy, return to use is common within the first 6 to 12 months postpartum. Return to use often leads to fatal overdose, underscoring the need for access to medication for opioid use disorder (Krans & Patrick, 2016). During the postpartum period, the challenge of adhering to treatment is often complicated by the intensive physical needs of infants and the critical need for maternal bonding (Prince & Ayers, 2022).

People with a SUD may also develop a co-occurring mental health disorder during their lifetime, and vice versa (National Institute of Mental Health, 2021). Among women who have a SUD and co-occurring mental health disorders, a diagnosis of posttraumatic stress disorder, anxiety, postpartum depression, and other mood disorders is more prevalent than among women who do not use substances (SAMHSA, 2009). Although national attention to SUDs has increased in recent years, barriers to receiving SUD care persist. Pregnant women with SUDs may avoid seeking prenatal care and other preventive health care services due to perceived social stigma or discrimination, inaccessibility of services, inequities in screening practices, and fear of prosecution or loss of infant custody (ACOG, 2017; Hand et al., 2017; Jackson & Shannon, 2012).

State health agencies, and more specifically, Title V programs, have the public health authority and influence as well as expertise in serving perinatal people, which makes them ideal partners to collaborate with substance use disorder treatment and mental health agencies, Medicaid, local health departments, universities, and others to create sustainable approaches to improving care for perinatal people with SUD.

### Funding Opportunity Through HRSA’s Maternal and Child Health Bureau

In response to the rising incidence of SUDs among perinatal people, HRSA’s MCHB released a call for proposals for innovative state-level policy initiatives that improve access to quality health care for MCH populations, with an emphasis on perinatal people with SUD. HRSA awarded five-year cooperative agreements (from 2018 to 2023) to four organizations, including AMCHP and ASTHO. With their closely aligned constituencies, AMCHP and ASTHO formed a partnership, called PRISM - “Promoting Innovation in State and Territorial MCH Policymaking.”

## The PRISM Team

AMCHP’s mission is to lead and support programs nationally to protect and promote the optimal health of women, children, youth, families, and communities. AMCHP’s core membership is composed of senior leadership from each state and territorial Title V/MCH program. Since its founding in 1950, AMCHP has served as a national resource, partner, and advocate for state public health leaders and others working to improve maternal and child health public health systems. AMCHP builds the capacity of diverse state and territorial MCH leadership teams to use tools to advance innovative solutions to improve women’s access to care for mental health and substance use disorders.

ASTHO’s mission is to support, equip, and advocate for state and territorial health officials in their work of advancing the public’s health and well-being. As a nonprofit organization, ASTHO is committed to supporting the work of state and territorial public health officials and their agencies and furthering the development and excellence of public health policy nationwide. ASTHO’s Maternal and Child Health team engages state and territorial health officials, and their agencies, to modify or enhance existing state-level policy initiatives that improve quality health care, including treatment for mental health and substance use disorders, for MCH populations.

### Overview of the PRISM Learning Community

The PRISM Learning Community model was comprised of five components: (1) establishing cross-agency teams, (2) developing action plans, (3) participating in virtual and in-person learning opportunities, (4) facilitating peer-to-peer sharing, and (5) providing technical assistance. Participating states and jurisdictions were selected through a request for applications process. The learning community included two consecutive cohorts, and state teams are actively engaged in the learning community for a minimum of 12 months.

#### Cross-Agency State Teams

As part of their application, jurisdictions were required to assemble a team of multiagency stakeholders, which included the state/territorial health official or designee, Title V/MCH director, substance use/behavioral health director and a Medicaid official. States were also encouraged to include representatives from the provider community, perinatal quality collaboratives, local health departments and community-based organizations. Connecting stakeholders across multiple agencies, reducing barriers to communication, and developing shared goals was instrumental to the success of participating jurisdictions.

#### Action Plan Development

When they first began actively participating in the learning community, state teams engaged in facilitated discussions with PRISM staff to develop action plans. Using a root cause analysis or similar tool, teams identified challenges and barriers to implementing policy or programmatic approaches to addressing the needs of perinatal people with SUDs. Teams were also encouraged to identify topical areas in which they may have the highest collective impact and in which they may have existing programs or policies. Once barriers and challenges had been identified, teams developed one or two SMART^1^ goals with associated strategies, action steps, and assigned roles/responsibilities. After goals were determined, action plans were developed to guide technical assistance requests and record jurisdictional progress.

#### Peer-to-Peer Sharing

The learning community model is structured to facilitate peer-to-peer sharing across jurisdictions. During in-person and virtual meetings, teams shared successes, lessons learned, and resources. Additionally, teams engaged in affinity group sessions during which they shared barriers to implementation and emerging issues with individuals who performed the same role. The PRISM team facilitated connections between jurisdictions and provided them a dedicated space to learn from one another.

#### Virtual and In-Person Programming

Learning community teams participated in virtual and in-person programming, which included policy academies, webinars, state share calls, and closeout meetings. During the policy academy, teams gathered to learn from federal and national partners and peers as well as engage in breakout learning sessions, action planning sessions, and panel presentations. Through webinars, the learning community teams had an opportunity to showcase promising, evidence-based, or evidence-informed best practices; learn from subject matter experts in the field; and share relevant resources. Topics included, but were not limited to, maternal mental health; SUD payment models; behavioral health integration and service co-location; universal screening and testing; nontraditional providers, including doulas and peer-recovery coaches; clinical guidelines [e.g., Alliance for Innovation on Maternal Health (AIM) safety bundles]; health equity; and impacts of COVID-19. The PRISM webinars were open to the public, allowing them to serve as an important tool for educating the cohort teams and the MCH field at large about innovations in addressing perinatal SUD.

#### Technical Assistance

ASTHO and AMCHP provided a continuum of technical assistance to support jurisdictional teams in developing and implementing action plan goals. The PRISM project’s technical assistance framework offered on-demand technical assistance, trends analysis and research, and technical assistance or expert consultation. Examples of technical assistance include connections to other jurisdictions and/or subject matter experts, policy scans and compilation of existing resources and tools. Additionally, AMCHP and ASTHO convened a stakeholder committee comprised of clinicians, public health professionals, and academics with expertise in perinatal SUD. Throughout the learning community, the stakeholder committee provided technical assistance resources, which included serving as webinar speakers, providing feedback on PRISM documents, and supporting state needs.

## Methods

**Fig. 1:**
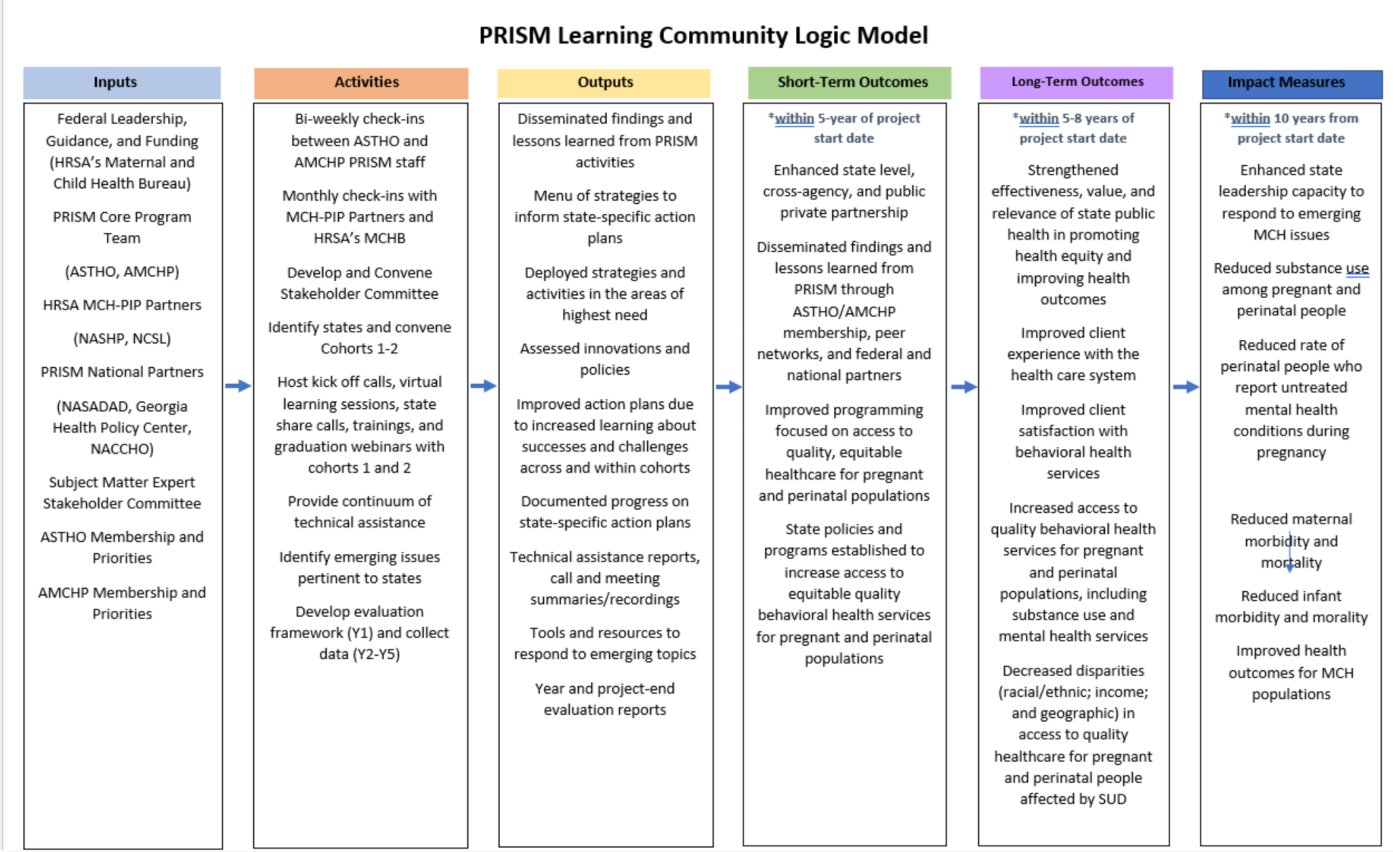
PRISM logic model

To assess the impact of the learning community model on programmatic and policy success, the PRISM team utilized a comprehensive evaluation framework, informed by the project’s logic model (Fig. 1). The evaluation framework followed a mixed methods approach, which included document reviews; pre-, midpoint, and post-surveys; and interviews. Surveys were electronically distributed to each participating jurisdiction team and captured longitudinal data on the team’s strategies, implementation efforts, policy environments, stakeholder engagement, and team’s effectiveness. The post-survey captured additional information on the overall experience and changes as a result of participating in the learning community. Surveys included closed and open-ended questions. A descriptive analysis was conducted for all surveys. To investigate changes over time between the pre-survey and post-survey, the descriptive analysis was restricted to only those participating jurisdictions that responded to both the pre- and post-survey. Interviews with participating jurisdiction teams were conducted at the end of their active engagement period via Zoom. Interviews captured more in-depth information on similar topics as the post-survey. As part of the document review, the PRISM team conducted a thematic analysis of state action plans and technical assistance to better understand the areas of greatest need, which would in turn, inform programming and sustainability planning. This manuscript is not based on clinical study or patient data, therefore IRB approval was not required.

## Results

### Evaluation Findings

Cohort 1 included Arkansas, Iowa, New Mexico, South Carolina, Washington state, and the Commonwealth of the Northern Mariana Islands. Cohort 1 was actively engaged from February 2019 through January 2021 (24 months). The Cohort 1 evaluation concluded in mid-2021. All jurisdictions responded to the pre-survey, four responded to the post-survey, and four participated in interviews. All Cohort 1 jurisdictions are represented in the technical assistance analysis. Cohort 2 jurisdictions included Indiana, Missouri, and Nevada. Cohort 2 was actively engaged from May 2021 through September 2022 (16 months). The Cohort 2 evaluation is ongoing; therefore, only results from the thematic analysis of technical assistance requests include Cohort 2 results. All other results presented are from Cohort 1 only.

### Cohort 1 Learning Community Engagement

Participation in the PRISM Learning Community provided access to space and guidance to create partnerships within teams and jurisdictions. The intent of the learning community was to support teams in developing and sustaining collaborative relationships with stakeholders. In the pre-, post-surveys, and interviews, responding teams indicated that partnerships across agency teams were strong and showed sustainability throughout the duration of the 15-month long cohort.

- *“Our [PRISM] task force has been sustained over three years and has built strong partnerships among the members.” (Cohort 1 participant)*.

While the learning community intended to shift indicators within health agencies related to “establishing roles and responsibilities that cross organizational boundaries” and “having regular effective communication,” the pre- and post-surveys revealed decreases in most teams’ self-reported effectiveness in these areas (three of four jurisdictions indicated they decreased their effectiveness, respectively). Teams reported that the challenges of the COVID-19 pandemic was a key reason they became less effective than at the start of the learning community.

- *“Unfortunately, communication has nearly stopped because of COVID.” (Cohort 1 participant)*.

Among the four participating jurisdiction teams that responded to the post-survey, all agreed the PRISM Learning Community was valuable to their work, increased their jurisdiction or agency’s capacity, increased their knowledge related to their area of public health practice, increased the number of connections with peers from other health agencies, and helped improve cross-sector collaborations. Teams reported that in-person and virtual programming, along with technical assistance, supported them in accomplishing goals, bringing together national partners, creating networking opportunities, increasing connections, and helping centralize the conversation around state priorities and data. The programming and technical assistance also allowed states to reflect on what other states were able to accomplish with Medicaid funds.

- *“I think [the PRISM Learning Community] helped with collaborative learning, learning from what other states have done and seen, what they were doing.” (Cohort 1 participant)*.

## Key Technical Assistance Themes in PRISM Jurisdictions’ Perinatal SUD Efforts

AMCHP and ASTHO staff analyzed key themes across state team action plans and technical assistance requests to inform sustainability planning and future learning opportunities. Four key themes in jurisdictions’ response to perinatal SUD emerged. They included the importance of (1) leveraging multisector collaborations, (2) supporting equitable screening practices, (3) developing the perinatal behavioral health workforce, and (4) exploring opportunities to enhance Medicaid coverage for perinatal SUD.

### Leveraging Multisector Collaborations

A foundational activity of the PRISM Learning Community was to establish multisector collaborations across various agencies and systems. Perinatal people with SUD interact with a range of stakeholders, including state and local health agencies, child welfare agencies, law enforcement, social service providers, Medicaid agencies, and hospital and medical systems. Multisector collaboration is increasingly necessary to provide comprehensive, coordinated, and equitable approaches to addressing SUD in perinatal people. One example of cross-sector collaboration is the Washington state PRISM team’s project, which brought together state agencies, housing advocacy organizations, and nonprofits to address the housing needs of families affected by the opioid epidemic and ensure low-barrier access to safe and affordable housing. PRISM team development and action plan strategies were largely rooted in and supported by this type of multisector collaboration.

### Supporting Equitable Screening Practices

Cohort teams expressed interest in best practices and policy opportunities to implement and incentivize universal verbal screening for substance use during perinatal health care visits. Although people of color are no more likely than white people to use illicit substances during pregnancy, they are far more likely to be screened for substance use and reported to criminal justice and child welfare systems for substance use than their white counterparts (Harp & Bunting, 2020). The American College of Obstetricians and Gynecologists (ACOG) Committee on Obstetric Practice and the American Society of Addiction Medicine recommend universal Screening, Brief Intervention, and Referral to Treatment (SBIRT) to ensure equitable screening occurs for all perinatal people (ACOG, 2017). SBIRT is an evidence-based approach to early intervention and treatment for people who use substances or who are at risk of developing substance use disorders (SAMHSA, 2022). The Iowa PRISM team focused its efforts on increasing SBIRT usage among clinicians serving pregnant individuals through Title V. Through a partnership between Iowa’s Children’s Justice Bureau, the Bureau of Substance Abuse, the Title V MCH program, and substance use treatment providers, SBIRT and Naloxone training was provided to Title V nurses, social workers, and nurse practitioners. All perinatal people receiving direct services from state or local Title V programs are now screened for SUD and referred to a specialty SUD treatment provider as needed. Throughout the PRISM project, resources were developed and shared to reduce punitive screening policies and practices and promote supportive, nonjudgmental strategies.

### Developing the Perinatal Behavioral Health Workforce

Screening alone is not enough to ensure that individuals with perinatal SUD receive an accurate diagnosis, referral to a specialist, and family-based treatment (Byatt et al., 2018). Jurisdictions indicated that programs and policies need to build workforce skills and capacity in addressing perinatal substance use. Workforce-related needs included promoting and incentivizing SBIRT use, integrating behavioral health care into perinatal care settings; leveraging the peer recovery workforce; and helping health care providers develop confidence and competency to screen, refer, and treat individuals with perinatal behavioral health concerns. One strategy to help jurisdictions address workforce challenges and better coordinate mental health care and SUD is to implement provider-to-provider teleconsultation lines, an evidence-based model to engage perinatal psychiatrists in consultation with providers such as OB-GYNs and primary care doctors. Teleconsultation allows different providers working with perinatal individuals to consult with psychiatrists who specialize in perinatal mental health. The PRISM team convened Cohort 2 teams to learn from an existing perinatal teleconsultation model, known as Massachusetts Child Psychiatry Access Program (MCPAP) for Moms, and facilitate peer-to-peer exploration of opportunities for program replication (Wichman et al., 2019).

### Exploring Opportunities to Enhance Medicaid Coverage for People with Perinatal SUD

Throughout the PRISM project, teams explored a variety of mechanisms to enhance Medicaid benefits and expand coverage for individuals with perinatal SUD. Jurisdictions expressed interest in adopting SBIRT into Medicaid programs as a cost-effective approach to early identification and intervention. If SBIRT billing codes are allowable within a state’s Medicaid system, providers can be compensated for their time; however, often restrictions are placed on provider types allowed to bill for SBIRT. Examples of Medicaid coverage for SBIRT were curated and shared with the learning community in an issue brief (AMCHP et al., 2020).

Given the growing need for perinatal behavioral health care, the learning community also explored opportunities to expand the behavioral health workforce through Medicaid coverage for nontraditional health care providers, including peer support specialists, doulas, and community health workers. Nationally, these nontraditional health care providers are not yet widely available as a covered Medicaid benefit; however, several states have successfully launched their own Medicaid coverage of some or all of these providers (Medicaid, 2019). PRISM programming and resources were developed to support the learning community’s focus on this topic and prominently feature doulas and peer recovery specialists to share their experiences and policy recommendations. Participants in the learning community reported that PRISM programming helped them learn about and reflect on what other states and jurisdictions were able to accomplish with Medicaid funds.

Overall, PRISM teams expressed a need to develop a more comprehensive understanding of the existing landscape of state and local perinatal SUD services, gaps, and opportunities to strengthen systems. Many teams requested technical assistance to explore strategies and policies from other jurisdictions related to their goals. These exploratory activities underscore the importance of learning communities in supporting policy development through state-to-state information sharing, access to subject matter expertise, and tailored resource development.

### Barriers to Perinatal SUD Efforts Across Jurisdictions

Barriers to advancing perinatal SUD policies and programs were identified through the learning community’s assessments. The most prominent barrier faced by jurisdictions was the competing demands placed on health agency staff and partners due to COVID-19. As early as January 2020, state and territorial health agency staff were reassigned to COVID-19 response activities, which limited staff time and resources to devote to PRISM projects. PRISM teams also reported a lack of sustainable funding for perinatal substance use disorder activities as a challenge to their overall efforts.

Notably, the stigma surrounding perinatal substance use was commonly cited as a challenge to providing high-quality, continuous care to pregnant and parenting people. It is well documented that people with SUD are at risk of stigmatizing and punitive interactions with health and social service providers, which frequently prevents people from seeking care or continuing their treatment (Trainor, 2022; Nichols et al., 2021; Stone, 2015). At a structural level, when policymakers stigmatize perinatal substance use, state and local health departments can have limited ability to provide pregnant and parenting people with the full range of judgment-free prevention, treatment, and harm reduction services (NCSACW, 2022). Addressing these challenges will require systemic changes to ensure that federal and state policies support, rather than hinder, public health best practices to improve outcomes for perinatal people who use substances and their families.

## Discussion

From 2018 to 2023, the PRISM Learning Community supported nine jurisdictions in advancing programs and policies supporting perinatal people with SUD. Through peer-to-peer learning and virtual programming, participants reported increased knowledge of perinatal SUD, strengthened interagency and cross-sector collaboration, and progress on action plan activities and goals. The four key priorities that emerged from the team’s action plans and technical assistance requests represent critical areas for future programming and technical assistance activities. As noted previously, these priorities are as follows:

- Leveraging multisector collaborations.
- Supporting equitable screening practices.
- Developing the perinatal behavioral health workforce.
- Exploring opportunities to enhance Medicaid coverage for perinatal SUD.

The demands of the COVID-19 pandemic placed unprecedented strains on state and local health departments staffing and resources. This situation created a significant barrier to team engagement and resulted in delays in completing the learning community action plans. The AMCHP and ASTHO teams shifted from in-person convenings to virtual platforms, which potentially compromised full engagement and participation. When facilitating action planning sessions with Cohort 2, AMCHP and ASTHO ensured that the goals were impactful and attainable, considering the ongoing need to respond to the COVID-19 pandemic. Despite the pandemic, teams expressed a need to sustain momentum related to their goals and activities.

To advance care and treatment for perinatal people with SUD, teams identified several programmatic and policy opportunities. First, they identified the importance of flexible funding to sustain activities related to perinatal SUD, especially when resources are diverted to emerging and urgent public health issues. In addition, they found that providing culturally competent, person-centered preventive care, treatment, and harm reduction services is key to reducing stigma associated with perinatal SUD. Furthermore, programs and policies should focus on ensuring health and racial equity. Finally, a cross-agency, cross-sector approach is critical to the uptake and advancement of care and treatment for perinatal people with SUD. Coordinated interventions at the local, state, and federal levels across disciplines could significantly improve perinatal mental health and SUD outcomes for MCH populations.

The PRISM team acknowledges two limitations of the analysis. The data collected from participants were self-reported. However, during interviews, respondents were asked to provide additional context and examples related to reported changes in survey metrics. In addition, ASTHO staff conducted the key informant interviews and interpreted the data; therefore, staff potentially influenced the participants’ responses or interpreted the information with some bias.

## Conclusion

Findings from the PRISM Learning Community emphasize the need for improved quality care and treatment for perinatal people with SUD. A strengthened, coordinated public health response is necessary to address barriers to access, including stigma, financing and reimbursement, workforce capacity, and competing public health issues. PRISM Learning Community successes can serve as a model for states and territories seeking to improve the health and well-being of families in their own jurisdictions.

## Data Availability

N/A.

## Footnotes

1 SMART is a mnemonic referring to goals that are Specific, Measurable, Achievable, Relevant, and Time-bound.

## Code Availability

N/A.
