# Correction to: Promoting Innovation in State and Territorial Maternal and Child Health Policymaking

**DOI:** 10.1007/s10995-023-03793-3

**Published:** 2023-10-25

**Authors:** Sanaa Akbarali, Ramya Dronamraju, Jessica Simon, Amani Echols, Stacy Collins, Betsy Kaeberle, Atyya Chaudhry

**Affiliations:** 1 Association of State and Territorial Health Officials, 2231 Crystal Drive, Suite 450, Arlington, VA, 22202 USA; 2 Association of Maternal and Child Health Programs, 1825 K Street NW, Suite 250, Washington, D.C, 20006 USA

**Correction to: Maternal and Child Health Journal**

10.1007/s10995-023-03779-1

The article ‘‘Promoting Innovation in State and Territorial Maternal and Child Health Policymaking’’, written by Sanaa Akbarali, Ramya Dronamraju, Jessica Simon, Amani Echols, Stacy Collins, Betsy Kaeberle and Atyya Chaudhry was originally published electronically on the publisher’s internet portal on 4 October 2023 without open access. With the author(s) decision to opt for Open Choice, the copyright of the article changed on 12 October 2023 to The Author(s) 2023 and the article is forthwith distributed under a Creative Commons Attribution 4.0 International License, which permits use, sharing, adaptation, distribution and reproduction in any medium or format, as long as you give appropriate credit to the original author(s) and the source, provide a link to the Creative Commons licence and indicate if changes were made. The images or other third-party materials in this article are included in the articles Creative Commons licence, unless indicated otherwise in a credit line to the material. If material is not included in the articles Creative Commons licence and your intended use is not permitted by statutory regulation or exceeds the permitted use, you will need to obtain permission directly from the copyright holder. To view a copy of this licence, visit http://creativecommons.org/licenses/by/4.0.

